# Visual Feedback–Based Rehabilitation Improves Gait Recovery After Total Hip Arthroplasty: A Randomized Controlled Clinical Trial

**DOI:** 10.2106/JBJS.OA.26.00065

**Published:** 2026-10-15

**Authors:** Riccardo Spanò, Antonio Ammendolia, Nicola Marotta, Alessandro de Sire, Maria Girolama Raso, Simone Carozzo

**Affiliations:** 1 S. Anna Institute, Crotone, Italy; 2 Department of Physical and Rehabilitation Medicine, AOU Renato Dulbecco / Magna Graecia University (Mater Domini), Catanzaro, Italy

## Abstract

**Background:**

This randomized controlled trial investigated whether adding a visual feedback–based (VFB) rehabilitation program to standard care could enhance gait performance and pelvic kinematics in patients recovering from total hip arthroplasty (THA).

**Methods:**

Inpatients aged 55 to 80 years who underwent unilateral THA by a lateral approach were randomized to either a VFB group or a control group. The VFB group received 30 minutes of technology-assisted visual feedback training using TecnoBody systems plus 30 minutes of conventional physiotherapy daily, while the control group received one hour of conventional therapy alone, both 5 days per week for 6 weeks. Gait and pelvic kinematics were assessed using the G-WALK inertial sensor at baseline and after 6 weeks, alongside clinical measures for pain, functional independence, and ambulation capacity.

**Results:**

Both groups showed significant improvements on clinical scales, with no meaningful differences between them. However, the VFB group demonstrated significantly greater gains in key gait and kinematic parameters, including cadence, speed, symmetry, pelvic tilt, and pelvic obliquity, with multivariate analysis confirming a distinct and more favorable post-treatment kinematic profile.

**Conclusion:**

Incorporating visual feedback–based training into postoperative rehabilitation after THA leads to superior improvements in gait quality and pelvic control compared with conventional therapy alone, supporting its clinical integration in standard physiotherapy programs.

**Level of Evidence:**

Therapeutic Level I. See Instructions for Authors for a complete description of levels of evidence.

## Introduction

Hip osteoarthritis is one of the most common degenerative musculoskeletal diseases in clinical practice, with a prevalence of over 500 million worldwide, as documented in large epidemiological analyses of osteoarthritis burden^1^. In the initial stages conservative treatments, including drugs and physical therapies, are preferred because of their early efficacy and role in slowing decline of hip OA^2,3^. This pathology progressively impairs functional ability: Severe gait changes are often observed in advanced disease, prompting indication for prosthetic surgery, as highlighted in studies exploring gait determinants and compensatory patterns in hip osteoarthritis^4^.

Despite the efficacy of total hip arthroplasty (THA)—which exceeds 1,000,000 annual procedures and significantly improves pain and quality of life^5^—preoperative gait alterations are not corrected by surgery alone. Without structured rehabilitation, these impairments may worsen due to muscle atrophy and altered motor patterns^6,7^.

Furthermore, 15% to 30% of patients report suboptimal outcomes. Recovery varies by surgical approach; for instance, lateral approaches can cause hip abductor weakness and an increased Trendelenburg’s sign^8-10^. Consequently, targeted rehabilitation is essential to restore functionality, especially in patients with poor recovery curves^7,11^.

To evaluate progress, traditional clinical scales (e.g., VAS, BI, and FAC) are widely used but remain subjective, operator-dependent, and influenced by patient mood^12-14^. Conversely, objective tools such as instrumented gait analysis and proprioceptive assessments provide quantifiable, reproducible data on gait and pelvic kinematics. Recent validations of inertial measurement units, such as the G-WALK system^15^, demonstrate their reliability in delivering precise, objective evaluations of postoperative recovery and prosthetic function.

Visual feedback–based rehabilitation (VFB) improves dynamic stability and gait recovery by real-time center-of-mass/load monitoring during proprioceptive and stepping tasks^16-18^. VFB integrates bottom-up sensory input with top-down cortical control to reinforce movement execution and postural control^19-22^.

For this reason, the aim of this randomized controlled trial was to evaluate the efficacy of an advanced motor rehabilitation protocol integrating an innovative VFB system for patients following THA, through instrumented kinematic gait analysis.

## Methods

Participants were recruited from the S. Anna Institute (Crotone) inpatient rehabilitation unit based on specific criteria (age 55-80 years, unilateral THA, and direct lateral approach) to ensure a homogeneous study population, aligning with prior clinical trials^7,23^. Exclusion criteria included inflammatory/infectious disease, BMI >30 kg/m^2^, neurological/musculoskeletal conditions affecting movement, sensory deficits, MMSE <24, and any contraindications to rehabilitation^9^.

Demographic and anthropometric data (height, weight, and BMI) were extracted at baseline (T0) using calibrated SECA instruments and cross-verified. The study adhered to the Declaration of Helsinki and Legislative Decree 196/2003, receiving local Ethics Committee approval and prospective registration in the ISRCTN registry (ISRCTN 53840608). All participants provided written informed consent.

No adverse events or protocol deviations occurred. Licensed physiotherapists supervised all sessions, monitoring patients for pain, fatigue, and hemodynamic stability. Conducted within the Italian National Health Service, this study used an inpatient subacute rehabilitation facility where patients typically complete a structured 4- to 6-week multidisciplinary program post-THA, contrasting with shorter acute inpatient stays and outpatient-focused models common in other healthcare systems.

Interventions began within 48 hours of admission^24^. Randomization (1:1) used a computer-generated sequence with sealed opaque envelopes for allocation concealment. The experimental group received a visual feedback (VFB) protocol using the TecnoBody system (Walker View 3.0 SCX, ProKin 252, and Digital-Wall). Each 30-minute VFB session—divided equally across the 3 devices—was immediately followed by 30 minutes of conventional therapy, performed 5 days/week for 6 consecutive weeks. Walker View 3.0 SCX is a sensorized treadmill optimizing gait speed, step symmetry, and load distribution; ProKin 252 is an electropneumatic platform using center-of-pressure trajectories to enhance dynamic balance, while Digital Wall is a virtual mirror system providing real-time 16-joint kinematic feedback. Thus, VFB progression required: (1) no compensatory trunk lean during load acceptance, (2) <10% variability in balance trajectories, (3) ≥80% target matching on the Digital Wall, (4) no pain exacerbation (>2 VAS points), and (5) therapist-verified readiness. The control group received only conventional rehabilitation (1-hour sessions, 5 days/week for 6 weeks). Both groups followed an identical conventional protocol: ROM exercises: hip flexion, extension, abduction, and rotation (2-3 sets of 10-15 repetitions); isometric strengthening: gluteus medius/minimus, quadriceps, and hip extensors (3 sets of 10-second holds); progressive resistance: elastic bands or ankle weights (2-3 sets of 10-12 repetitions) upon pain control/load tolerance; and last, for balance and gait: parallel bar ambulation, step training, and weight-shifting, progressed based on individual tolerance and movement quality.

Outcome measures were collected at baseline (T0) and 6 weeks (T1) by blinded, independent assessors in a separate clinical area to prevent treatment disclosure. Blinding applied to all outcomes. We performed: Visual Analog Scale (VAS), a 10-cm self-report line measuring subjective pain intensity from “no pain” to “worst possible pain”^12,14^; Barthel Index (BI), a 10-item activities of daily living independence measure and scores range 0 to 100 (higher means more independent)^13,25^; and Functional Ambulation Category (FAC), A 6-point scale (0-5) assessing walking ability from nonfunctional (0) to fully independent^5,26,27^.

VFB training progressed based on patient tolerance and recovery stage, increasing weekly in complexity, duration, and dynamic demands. Each session lasted 30 minutes, divided into three 10-minute interventions. First, the Walker View 3.0 SCX sensorized treadmill focused on gait speed regulation, step symmetry, and lower-limb load distribution, using an integrated 3D camera to provide real-time visual feedback on posture, load asymmetry, and joint range of motion (ROM) during walking. Next, the ProKin 252 electropneumatic balance platform engaged participants in 3 to 4 exercises in mono- and bipodalic stances. This component provided immediate visual feedback on center-of-pressure (COP) trajectories to target dynamic balance control, proprioception, and lower-limb joint ROM. Finally, the Digital Wall virtual mirror system involved 6 to 8 whole-body exercises to improve posture, strength, joint mobility, and balance, recognizing up to 16 body joints to deliver real-time kinematic feedback on movement accuracy.

### Statistical Analysis

Sample size was estimated with GPower, based on gait speed differences consistent with prior THA rehabilitation studies. With an alpha level of 0.05, statistical power of 0.80, and an effect size of 0.6, the required sample size was calculated as 40 participants per group. To account for potential dropouts, we recruited 45 participants per group, yielding a total sample of 90 individuals.

Data were analyzed using R (version 3.5.2; R Foundation for Statistical Computing, Vienna, Austria). Between-group comparisons were performed with Student’s t-tests; within-group temporal changes were evaluated with repeated-measures ANOVA. A multiple factor analysis (MFA)^14,28^ was conducted using the FactoMineR package with visualization via factoextra^29^. Importantly, these multivariate and clustering procedures were conducted as exploratory, *post hoc* analyses and were not prespecified as primary inferential end points in the original trial protocol. MFA was selected over simpler multivariate approaches, such as MANOVA, due to the structured and heterogeneous nature of our data set, which contains mixed blocks of variables (i.e., categorical clinical scores and continuous multidimensional kinematic metrics). Unlike MANOVA, which tests group mean differences under strict parametric constraints, MFA allows for the simultaneous integration of distinct data blocks, normalizing their weights to prevent variables with larger dimensions from disproportionately dominating the total variance.

Hierarchical clustering and K-means clustering were applied to participant MFA scores to identify subgroups; dimension-reduction techniques and the gap-statistic method supported cluster selection and interpretation^30^. Cluster validity was assessed using R^2^, Akaike Information Criterion (AIC), and silhouette scores, computed in JASP (version 0.16).

## Results

Of the 112 patients assessed for eligibility, 22 were excluded (17 did not meet inclusion criteria and 5 refused). Ninety patients provided informed consent, were randomized into 2 groups, and completed the study with no losses to follow-up, reflecting strong feasibility, as depicted in Fig. 1. At baseline (T0), no statistically significant differences in demographic or clinical characteristics were observed between groups, supporting balanced randomization (Table 1).

**Fig. 1:**
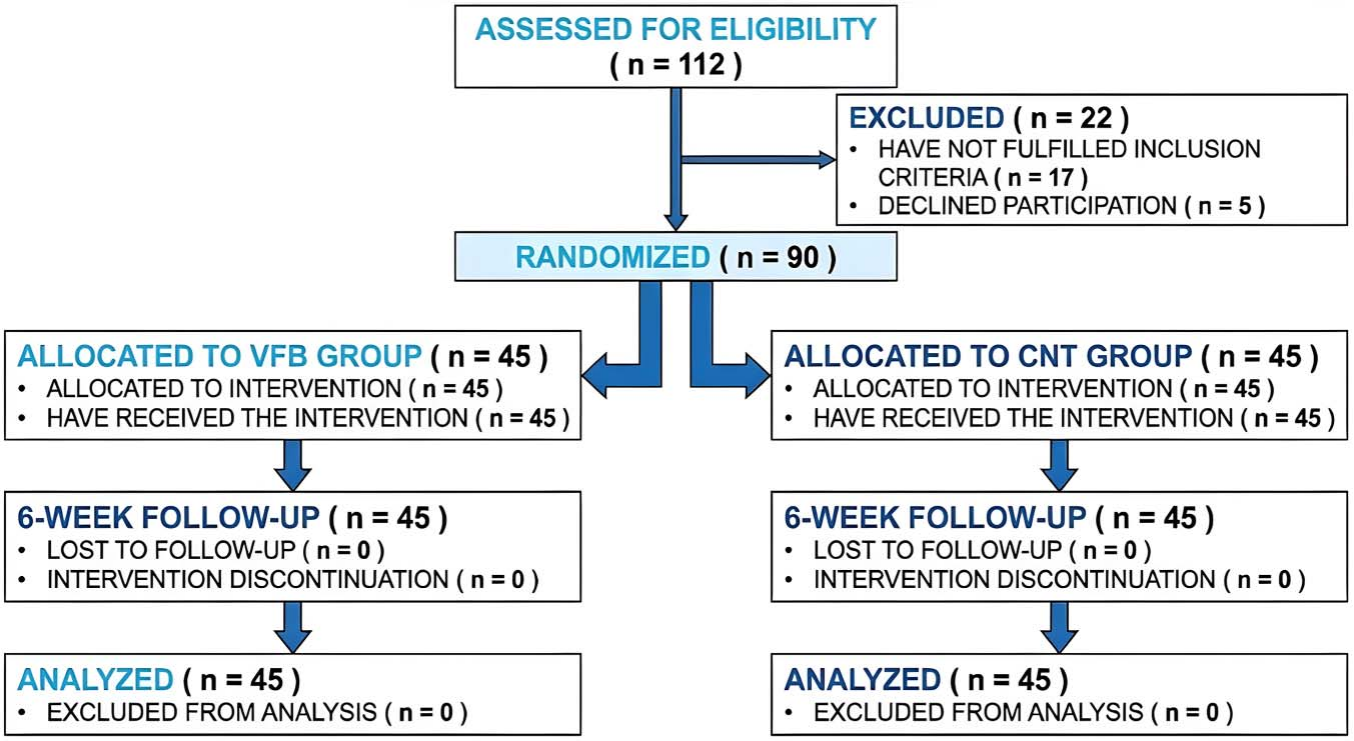
CONSORT flow diagram of participant progression through the study.

**Table 1:** Baseline Characteristics of Participants

		Mean	SD	p
BMI (kg/m^2^)	CNT (n = 45)	25.95	2.51	0.518
	EXP (n = 45)	26.32	3.03	
AGE (years)	CNT (n = 45)	69.71	11.41	0.183
	EXP (n = 45)	66.64	12.79	
Hospitalization (days)	CNT (n = 45)	34.17	10.69	0.504
	EXP (n = 45)	35.42	9.84	

BMI = body mass index, CNT = control (control group), EXP = experimental (experimental group), n = number of participants in the group (sample size), p-value = probability value, and SD = standard deviation.

After rehabilitation, both groups showed significant and similar improvements measured by clinical scales (see Table 2)^12-14^. The experimental (EXP) group reduced their VAS deficit from 45.78 ± 14.7 to 10.0 ± 9.29 and the control (CNT) group from 46.22 ± 16.41 to 10.88 ± 9.0. The Barthel index improved from 43.86 ± 18.05 to 83.8 ± 14.77 for CNT and from 42.68 ± 17.53 to 86.6 ± 9.84 for EXP (p < 0.001).

**Table 2:** Differences in Clinical and Kinematic Outcome Measures

		T0		T1		ΔT0-T1
		Mean	SD	Mean	SD	p
Barthel index	CNT	43.86	18.05	83.80	14.77	0.001
	EXP	42.68	17.53	86.60	9.84	0.001
Between group p		0.75		0.29		
FAC	CNT	1.06	1.01	3.77	0.87	0.001
	EXP	0.80	0.62	3.97	0.49	0.001
Between group p		0.135		0.187		
VAS	CNT	46.22	16.41	10.88	9.00	0.001
	EXP	45.77	14.69	10.00	9.29	0.001
Between group p		0.89		0.64		
CADENCE (steps/min)	CNT	61.22	18.69	82.11	21.03	0.001
	EXP	59.93	15.62	91.18	14.43	0.001
Between group p		0.724		0.019		
GAIT SPEED (m/s)	CNT	0.53	0.16	0.74	0.20	0.001
	EXP	0.52	0.15	0.83	0.16	0.001
Between group p		0.933		0.035		
Cycle duration (s)	CNT	2.35	0.68	1.58	0.40	0.001
	EXP	2.47	0.63	1.45	0.31	0.001
Between group p		0.379		0.104		
Cycle length (m)	CNT	1.10	0.21	1.20	0.21	0.625
	EXP	1.11	0.14	1.21	0.12	0.633
Between group p		0.851		0.771		

T0 = baseline, T1 = follow-up, CNT = control group, EXP = experimental group, FAC = functional classification of gait, and VAS = Visual Analog Scale for pain.

Data are shown as means ± standard deviations. Statistical threshold * = p < 0.005.

Conversely, gait analysis revealed significant postrehabilitation improvements in the EXP group compared with CNT in 5 indices (Table 3)^16-18^: cadence (p = 0.019; EXP: 91.18 ± 14.43 vs CNT: 82.11 ± 21.03), gait speed (p = 0.035; EXP: 0.83 ± 0.17 vs CNT: 0.74 ± 0.20), symmetry (p = 0.025), pelvic tilt (p = 0.044), and obliquity (p = 0.017).

**Table 3:** Differences in Pelvic Kinematic Parameters

Pelvic motion		T0		T1		ΔT0-T1
		Mean	SD	Mean	SD	p
Symmetry index	CNT	72.23	13.36	81.30	14.03	0.005
	EXP	71.11	12.55	86.69	7.38	0.001
Between group p		0.681		0.025		
Propulsion index	CNT	3.47	1.22	5.45	1.68	0.001
	EXP	3.36	1.24	5.44	1.46	0.001
Between group p		0.677		0.986		
TILT (°)	CNT	66.74	15.14	78.83	17.30	0.032
	EXP	67.15	15.86	84.81	9.30	0.001
Between group p		0.9		0.044		
Obliquity (°)	CNT	43.72	22.1	55.07	21.78	0.011
	EXP	47.83	17.14	65.57	18.98	0.002
Between group p		0.327		0.017		
Rotation (°)	CNT	69.13	17.82	77.98	13.81	0.006
	EXP	66.11	21.43	83.90	13.76	0.001
Between group p		0.469		0.045		

T0 = baseline, T1 = follow-up, CNT = control group, and EXP = experimental group.

Data are shown as means ± standard deviations. Statistical threshold * = p < 0.005.

Using linear regression, pelvic movement parameters were evaluated two-dimensionally on a Cartesian plane (Fig. 2). At T0, patients were arranged homogeneously (Fig. 2-A). Fig. 2-B showed how individual variables weighted on the axes, while Fig. 2-C illustrated participant arrangement based on these weights to understand their influence^16-18^

**Fig. 2:**
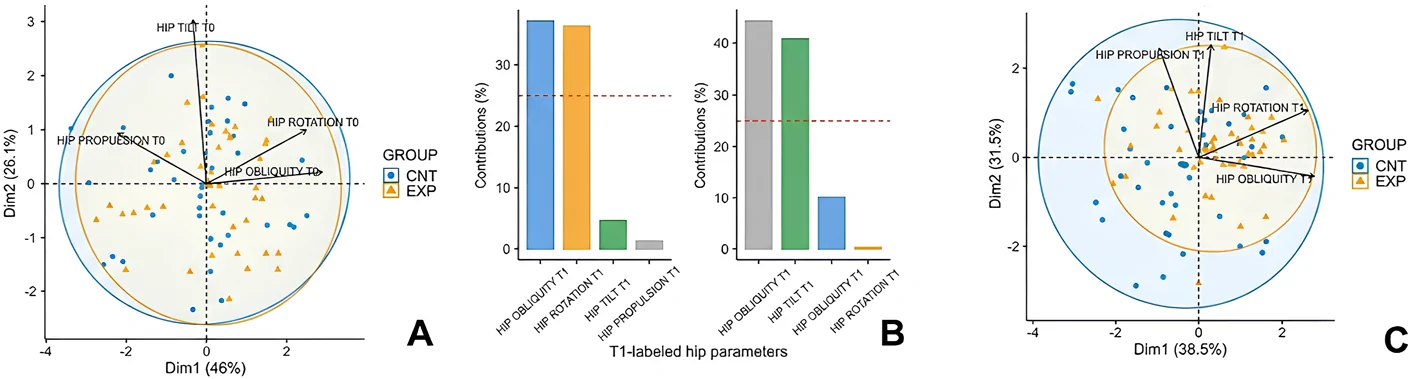
Multiple factor and PCA of hip kinematics at baseline (T0) and post-treatment (T1). **Fig. 2-A** At T0, overlapping ellipses confirm homogeneous baseline deficits between groups (CNT vs. EXP). **Fig. 2-B** Variable plots identify pelvic obliquity, rotation, tilt, and propulsion as the main drivers of gait variance. **Fig. 2-C** At T1, the distinct separation and narrowing of ellipses demonstrate that visual feedback (EXP) induces a significantly superior, more symmetrical normalization of gait compared with CNT. CNT = conventional therapy, EXP = experimental (experimental group), and PCA = principal component analysis.

Therefore, hierarchical clustering identified a clear functional dichotomy: Cluster [X] (predominantly EXP) represents a high-functioning recovery profile driven by improved pelvic symmetry and gait economy, whereas Cluster [Y] reflects a conservative pathway with persistent asymmetries. Ultimately, active VFB positioned EXP participants in the upper right corner at T1, separating them from CNT patients, who remained near T0 positions. This shift is due to the positive influence of “rotation” and “obliquity” on the x-axis (Dim1) and “tilt” and “propulsion” on the y-axis (Dim2)^28,30-32^.

## Ethics Approval

The study was approved by the Comitato Etico di Catanzaro (Italy).

All participants gave written informed consent before data collection began. Study registration: ISRCTN53840608.

## Discussion

This randomized controlled trial evaluated a novel visual feedback–based (VFB) rehabilitation protocol versus standard care after total hip arthroplasty (THA). While both groups improved on subjective clinical scales (BI, VAS, and FAC), no between-group differences emerged, matching known sensitivity limitations of self-reported metrics^12-14^. Conversely, the VFB group showed superior improvements in kinematic gait parameters, proving objective metrics capture subtle changes^15^. Marked symmetry index improvements align with the literature showing VFB enhances load distribution^16-18^, while faster walking speed predicts better long-term mobility^33^, ultimately facilitating faster gait normalization than conventional therapy alone^5,34^.

The clinical relevance of these gait speed differences requires careful interpretation. The between-group difference at T1 was 0.09 m/s, slightly below the commonly reported minimal clinically important difference (MCID) for postoperative THA populations (0.10-0.14 m/s). This suggests these early changes may not yet have reached a threshold perceived by patients as functionally meaningful. However, a recent knee arthroplasty study found a gait velocity–specific MCID of approximately 0.067 m/s, suggesting thresholds in arthroplasty populations may be lower than the classic 0.10 m/s^35^.

Significant gains occurred in pelvic kinematics (obliquity, tilt, and rotation) within the VFB group. Pelvic motion depends highly on hip abductor strength and surgical approach^8,9,35^. Real-time visual cues enhance proprioceptive acuity and neuromuscular control, reducing postural uncertainty, promoting corrective motor strategies, and enhancing the reconditioning of hip stabilizers^19,21,22^.

Exploratory multivariate analyses (MFA and clustering) illustrated distinct post-treatment profiles. Experimental participants tended to align with dimensions driven primarily by improved pelvic control and symmetry. This maps mixed clinical and kinematic data into distinct patient profiles, proving VFB fosters a global biomechanical restructuring rather than merely modifying isolated parameters^28,31,32^. Clinically, recognizing these multidimensional recovery patterns can help clinicians identify suboptimal responders early and tailor biofeedback protocols to target specific pelvic-gait cluster phenotypes.

Overall, these findings advocate for integrating VFB into standard post-THA rehabilitation. Objective wearable-sensor analysis—such as G-WALK—offers a sensitive framework to quantify these improvements and guide individualized treatment. Future research should incorporate long-term follow-up to determine whether these early gains influence prosthetic survival or prevent degenerative changes over time.

This RCT is not free of limitations. First, while the sample size (n = 90) is sufficient for Level I evidence regarding primary objectives, it is modest for high-dimensional multivariate generalization, requiring larger multicenter cohorts for validation. Second, exploratory multivariate analyses (MFA and clustering) lacked an a priori power calculation, risking overfitting; they should be interpreted strictly as hypothesis-generating. Third, no multiple-comparison corrections were applied to secondary gait and pelvic metrics, increasing Type I error risks. Fourth, assessment was restricted to the immediate post-treatment period^5^, leaving long-term retention of gait symmetry unknown. Finally, despite strict criteria, baseline heterogeneity—such as individual compensatory strategies, preoperative osteoarthritis severity, or minor surgical variations—could have subtly influenced kinetic and kinematic responses to visual feedback rehabilitation^8,9,35^.

## Conclusions

Visual feedback–based (VFB) rehabilitation combined with conventional therapy significantly improves gait speed, symmetry, and pelvic kinematics compared with standard care alone. These objective gains, undetected by traditional clinical scales, underscore the value of instrumental gait analysis after total hip arthroplasty (THA). These findings support early, individualized protocols to optimize biomechanics and prevent compensatory strategies. Surgically, the study exclusively included patients undergoing a direct lateral approach. This technique is associated with abductor muscle weakness and specific gait impairments (e.g., Trendelenburg patterns) that the VFB protocol directly targets. In addition, the 6-week assessment captures only early postoperative recovery. Future research with medium- to long-term follow-up is needed to determine whether these early advantages are sustained over 6 to 12 months. Finally, the study reflects the Italian subacute inpatient model (a structured 6-week stay), which differs from systems with shorter stays (7-14 days). Nevertheless, the VFB protocol’s modular design (30 minutes within a 60-minute session) suggests it can be feasibly adapted to shorter inpatient programs, outpatient settings, or home-based telerehabilitation.
